# Genome Sequences of *Listeria monocytogenes* Strains with Resistance to Arsenic

**DOI:** 10.1128/genomeA.00327-17

**Published:** 2017-05-11

**Authors:** Vikrant Dutta, Sangmi Lee, Todd J. Ward, Nathane Orwig, Eric Altermann, Dereje D. Jima, Cameron Parsons, Sophia Kathariou

**Affiliations:** a Department of Food, Bioprocessing and Nutrition Sciences, North Carolina State University, Raleigh, North Carolina, USA; b Center for Human Health and the Environment, North Carolina State University, Raleigh, North Carolina, USA; c Bioinformatics Research Center, North Carolina State University, Raleigh, North Carolina, USA; d USDA-ARS, Peoria, Illinois, USA; e Rumen Microbiology, AgResearch Grasslands, Palmerston North, New Zealand; f Riddet Institute, Massey University, Palmerston North, New Zealand

## Abstract

*Listeria monocytogenes* frequently exhibits resistance to arsenic. We report here the draft genome sequences of eight genetically diverse arsenic-resistant *L. monocytogenes* strains from human listeriosis and food-associated environments. The availability of these genomes will help elucidate the role of heavy-metal resistance in the ecology of *L. monocytogenes*.

## GENOME ANNOUNCEMENT

The facultative intracellular foodborne bacterial pathogen *Listeria monocytogenes* is well known for its propensity for heavy-metal resistance, specifically to cadmium and arsenic. Cadmium resistance is frequently plasmid-associated and encountered among isolates of diverse serotypes, being especially common in serogroup 1/2 (1–4). Nonpathogenic *Listeria* spp. can conjugatively transfer such resistance to *L. monocytogenes* (5). In contrast, arsenic resistance is primarily encountered among *L. monocytogenes* strains of serotype 4b and is chromosomally mediated (2, 6, 7). Genome sequencing of strain Scott A revealed a 35-kb chromosomal island (*Listeria* genomic island 2 [LGI2]), which includes genes for arsenic and cadmium resistance (6, 7). However, mechanisms mediating arsenic resistance in *L. monocytogenes* strains of diverse sources and genotypes remain poorly understood. Here, we present the whole-genome sequences of eight arsenic-resistant *L. monocytogenes* strains, including 6 strains of serotype 4b and 1 strain each of serotypes 1/2a and 1/2c. Serotype 4b strains included 3 strains from food or food-processing environments, i.e., F8027 (celery; multilocus sequencing typing-based clonal complex 315 [CC315]), BS-26 (environment swab; CC1), and FDA 100 (environmental swab, 1986; CC2) and 3 CC1 human clinical isolates, J2213, J3422, and J4600 (from 2003, 2005, and 2007, respectively) (8). Thus, most serotype 4b strains were members of hypervirulent clonal complexes (9), specifically CC1 (BS-26, J2213, J3422, and J4600) and CC2 (FDA 100). The serotype 1/2a strain 2012-0070 (CC14) was from a food-processing environment in North Carolina, USA (2012), while 2008-911 (serotype 1/2c; CC9) was implicated in human listeriosis in North Carolina in 2008.

The genomic DNA extracted with the DNeasy blood and tissue kit (Qiagen, Valencia, CA) was used to prepare the sequencing libraries on a Zephyr next-generation sequencing (NGS) workstation (PerkinElmer, Waltham, MA) using the NEBNext Fast DNA library prep set (New England BioLabs, Ipswich, MA). The Ion Torrent Personal Genome Machine was used for sequencing with an Ion 318 Chip version 2 and the Ion PGM 400 sequencing kit (Life Technologies, Inc., Grand Island, NY). Overall, 6,175,875 single reads were produced, with a median read length of 282 nucleotides (nt). Barcode sequences attached during the library preparation were used for sorting the reads, and the CLC Genomics Workbench 7.5.1 (CLC bio, Boston, MA) software was used (with default parameters) for quality trimming and *de novo* assembly. Assembly size ranged between ca. 2.6 and 2.8 Mbp, with an average coverage of 28.7 to 61.5×, and between 122 and 348 contigs were generated. Annotations were performed using the GAMOLA 2 and the NCBI Prokaryotic Genome Annotation Pipeline (10). Genome annotations identified 2,813 to 3,042 coding sequences, 5 to 10 rRNAs, and 59 to 65 tRNAs.

The availability of these genome sequences will further facilitate analysis of the roles of heavy-metal resistance in the ecology and adaptive physiology of *L. monocytogenes* from diverse sources and of diverse genotypes and serotypes.

### Accession number(s).

This whole-genome shotgun project has been deposited in DDBJ/ENA/GenBank under the accession numbers MPBE00000000, MLFL00000000, MNCA00000000, MNCB00000000, MNCC00000000, MNCD00000000, MNCE00000000, and MNCF00000000. The versions described in this paper are the first versions.

## References

[1] Lebrun M, Loulergue J, Chaslus-Dancla E, Audurier A 1992 Plasmids in *Listeria monocytogenes* in relation to cadmium resistance. Appl Environ Microbiol 58:3183–3186. 1444434 10.1128/aem.58.9.3183-3186.1992 PMC183070

[2] McLauchlin J, Hampton MD, Shah S, Threlfall EJ, Wieneke AA, Curtis GD 1997 Subtyping of *Listeria monocytogenes* on the basis of plasmid profiles and arsenic and cadmium susceptibility. J Appl Microbiol 83:381–388. doi:10.1046/j.1365-2672.1997.00238.x. 9351219

[3] Mullapudi S, Siletzky RM, Kathariou S 2008 Heavy-metal and benzalkonium chloride resistance of *Listeria monocytogenes* isolates from the environment of turkey-processing plants. Appl Environ Microbiol 74:1464–1468. doi:10.1128/AEM.02426-07. 18192428 PMC2258618

[4] Kuenne C, Voget S, Pischimarov J, Oehm S, Goesmann A, Daniel R, Hain T, Chakraborty T 2010 Comparative analysis of plasmids in the genus *Listeria*. PLoS One 5:e12511. doi:10.1371/journal.pone.0012511. 20824078 PMC2932693

[5] Katharios-Lanwermeyer S, Rakic-Martinez M, Elhanafi D, Ratani S, Tiedje JM, Kathariou S 2012 Coselection of cadmium and benzalkonium chloride resistance in conjugative transfers from nonpathogenic *Listeria* spp. to other listeriae. Appl Environ Microbiol 78:7549–7556. doi:10.1128/AEM.02245-12. 22904051 PMC3485730

[6] Briers Y, Klumpp J, Schuppler M, Loessner MJ 2011 Genome sequence of *Listeria monocytogenes* Scott A, a clinical isolate from a food-borne listeriosis outbreak. J Bacteriol 193:4284–4285. doi:10.1128/JB.05328-11. 21685277 PMC3147710

[7] Kuenne C, Billion A, Mraheil MA, Strittmatter A, Daniel R, Goesmann A, Barbuddhe S, Hain T, Chakraborty T 2013 Reassessment of the *Listeria monocytogenes* pan-genome reveals dynamic integration hotspots and mobile genetic elements as major components of the accessory genome. BMC Genomics 14:47. doi:10.1186/1471-2164-14-47. 23339658 PMC3556495

[8] Lee S, Ward TJ, Graves LM, Tarr CL, Siletzky RM, Kathariou S 2014 Population structure of *Listeria monocytogenes* serotype 4b isolates from sporadic human listeriosis cases in the United States from 2003 to 2008. Appl Environ Microbiol 80:3632–3644. doi:10.1128/AEM.00454-14. 24705322 PMC4054141

[9] Maury MM, Tsai YH, Charlier C, Touchon M, Chenal-Francisque V, Leclercq A, Criscuolo A, Gaultier C, Roussel S, Brisabois A, Disson O, Rocha EP, Brisse S, Lecuit M 2016 Uncovering *Listeria monocytogenes* hypervirulence by harnessing its biodiversity. Nat Genet 48:308–313. doi:10.1038/ng.3501. 26829754 PMC4768348

[10] Altermann E, Lu J, McCulloch A 2017 GAMOLA2, a comprehensive software package for the annotation and curation of draft and complete microbial genomes. Front Microbiol 8:346. doi:10.3389/fmicb.2017.00346. 28386247 PMC5362640

